# Improved diagnostic performance of insertable cardiac monitors by an artificial intelligence-based algorithm

**DOI:** 10.1093/europace/euad375

**Published:** 2024-01-03

**Authors:** Eliot Crespin, Arnaud Rosier, Issam Ibnouhsein, Alexandre Gozlan, Arnaud Lazarus, Gabriel Laurent, Aymeric Menet, Jean-Luc Bonnet, Niraj Varma

**Affiliations:** Implicity SAS, Paris, France; Implicity SAS, Paris, France; Jacques Cartier Private Hospital, Massy, France; Implicity SAS, Paris, France; Implicity SAS, Paris, France; Service de rythmologie interventionnelle, Clinique Ambroise Paré, Neuilly sur Seine, France; Service de rythmologie et Insuffisance Cardiaque, Centre Hospitalier Universitaire, Dijon, France; Département de Cardiologie, Groupe Hospitalier de l'Institut Catholique de Lille, Lomme, France; Implicity SAS, Paris, France; Department of Cardiovascular Medicine, Cleveland Clinic, Cleveland, OH, USA

**Keywords:** insertable cardiac monitor, implantable loop recorder, remote monitoring, artificial intelligence, machine learning, arrhythmia

## Abstract

**Aims:**

The increasing use of insertable cardiac monitors (ICM) produces a high rate of false positive (FP) diagnoses. Their verification results in a high workload for caregivers. We evaluated the performance of an artificial intelligence (AI)-based ILR-ECG Analyzer™ (ILR-ECG-A). This machine-learning algorithm reclassifies ICM-transmitted events to minimize the rate of FP diagnoses, while preserving device sensitivity.

**Methods and results:**

We selected 546 recipients of ICM followed by the Implicity™ monitoring platform. To avoid clusterization, a single episode per ICM abnormal diagnosis (e.g. asystole, bradycardia, atrial tachycardia (AT)/atrial fibrillation (AF), ventricular tachycardia, artefact) was selected per patient, and analyzed by the ILR-ECG-A, applying the same diagnoses as the ICM. All episodes were reviewed by an adjudication committee (AC) and the results were compared. Among 879 episodes classified as abnormal by the ICM, 80 (9.1%) were adjudicated as ‘Artefacts’, 283 (32.2%) as FP, and 516 (58.7%) as ‘abnormal’ by the AC. The algorithm reclassified 215 of the 283 FP as normal (76.0%), and confirmed 509 of the 516 episodes as abnormal (98.6%). Seven undiagnosed false negatives were adjudicated as AT or non-specific abnormality. The overall diagnostic specificity was 76.0% and the sensitivity was 98.6%.

**Conclusion:**

The new AI-based ILR-ECG-A lowered the rate of FP ICM diagnoses significantly while retaining a > 98% sensitivity. This will likely alleviate considerably the clinical burden represented by the review of ICM events.

**What’s new?**

- A new artificial intelligence (AI)-based ILR-ECG Analyzer™ (ILR-ECG-A) has been developed to lower the rate of false positive (FP) diagnoses in insertable cardiac monitors (ICM), while retaining a sensitivity of over 98%.
- The ILR-ECG-A algorithm reclassified 76% of the episodes that were originally identified as FP by the ICM as normal, thus reducing the workload for caregivers in verifying these false positives.
- The ILR-ECG-A retained a high sensitivity of 98.6% in detecting abnormal episodes, which makes it a reliable diagnostic tool for arrhythmias, including atrial tachycardia (AT) and atrial fibrillation (AF).
- The ILR-ECG-A algorithm uses machine learning to discern the characteristics of the ECG associated with each abnormality, thereby reducing the number of FP detections.
- The ILR-ECG-A is CE marked class I and Food and Drug Administration (FDA) cleared class II through a 510(k) submission.
- The ILR-ECG-A has the potential to alleviate the clinical burden associated with the review of ICM events, allowing caregivers to focus on patients who require urgent attention.

## Introduction

Follow up of recipients of implantable cardiac electronic devices has been facilitated by automatic remote monitoring (RM) which offers early event detection and enables, when needed, timely treatment.^1^ For example, insertable cardiac monitors (ICM) permit much longer monitoring of patients with suspected atrial fibrillation (AF) compared to usual care^2^ or standard 24-h ambulatory ECG recordings.^3^ ICMs are now widely and increasingly used in routine care and represent an important diagnostic instrument, most notably for cryptogenic strokes, unexplained syncope, palpitations, and a variety of arrhythmias, and particularly AF.^4,5^ However, adjudication of device events presents a huge workload to clinic staff. While ICM diagnostic algorithms differ among manufacturers and device models, clinical experience and peer-reviewed medical literature suggest consistently that these systems are highly sensitive to arrhythmias, but are vulnerable to a high rate of FP detections^6–8^ reported to be between 46% and 86%, depending on implant indication.^9^ Several solutions have been proposed to increase specificity. Among them, artificial intelligence (AI) may filter only the most actionable data to clinicians.^10^ These algorithms use large amounts of data to ‘train’ the computer by labelling each case according to one of many predefined abnormalities, allowing the machine to discern what characteristics of the ECG are associated with any given abnormality.

Here, we hypothesized that the ILR-ECG Analyzer™ (ILR-ECG-A) machine learning algorithm (Implicity™, Paris, France) designed to reclassify ICM recorded arrhythmias, would diminish the percentage of FP episodes i.e. increase specificity. This algorithm is CE marked class I and Food and Drug Administration (FDA) cleared class II.

## Methods

### Study design

Among 2643 recipients of Reveal LINQ, DX, or XT ICM entered in the Implicity database, we selected on 2020-11 546 patients (as was required by a preliminary sample size calculation) from 18 French and 13 US medical centres, with a random draw from a uniform distribution. Three different versions of the manufacturers’ ICM are then included in this study, in proportion close to what they were in this RM platform at the time of patient selection. The data for these patients had been transmitted between the ICM and the platform as part of the patients’ routine care. For these patients, we extracted all episodes transmitted from January 2014 to October 2020. For this study, the daily transmissions were anonymized, and the events were collected towards a retrospective analysis of the new ILR-ECG-A algorithm’s performance. Since this was a retrospective analysis of clinical data, this study was exempt from reviews and approvals by the institutional review boards of the participating institutions, in accordance with the European ‘General Data Protection Regulation’ (UE 2016/679). All patients had granted their written approval to contribute the data at the time of activation of RM. All data were de-identified to ensure the protection of personal health data, according to the regulation and French reference methodology.

### ICM-detected events

The three ICM algorithms can detect four main types of abnormal rhythms:

1. *Asystole*, defined as the absence of ventricular activity for a duration longer than a programmable value (1.5 s, 3 s, 4.5 s—the default is 3 s).
2. *Bradycardia*, defined as consecutive RR intervals (the intervals between two consecutive R-waves) above a programmable value (1.2 s, 1.5 s, 2.0 s—the default is 2.0 s).
3. *Atrial tachycardia* or *atrial fibrillation* (AT/AF) is detected using an automatic algorithm based on the R-R interval variability within a 2-min period. The differences between consecutive R-R intervals are displayed in a Lorenz plot. Pattern recognition is used to identify the AT and AF episodes; R-R intervals are highly irregular and uncorrelated during episodes of AF, whereas they are regular during episodes of AT. In addition, for Reveal LINQ specifically, a *P*-wave presence algorithm allows for the filtering of episodes that presents detectable *P*-wave in R-R intervals.
4. *Tachycardia* (Tachy) is defined as a ventricular rhythm with consecutive RR intervals below a programmable value (ranging from 0.27 s to 0.50 s—the default is 0.34 s).

These episodes are programmed at the time of ICM implantation and may be modified during follow-up. ICM has two means of recording the episodes:

1. The device analyzes the cardiac signal incessantly and records and stores the ECG when an episode is detected by the algorithm. Such episodes have durations ranging from 5 s to 2 min, with a sampling rate of 256 Hz for each of the three considered ICM devices.
2. The ICM captures an episode upon manual activation by the patient, who uses an assistant device or, in the latest models, a smartphone application. These episodes are usually activated when the patient experiences a symptomatic event, establishing a temporal relationship between symptoms and ICM recording.

While programming of the algorithm closely adapts the device to the patient’s needs, it is often insufficient to control the high rate of FP detections, mostly due to loss of R-wave amplitude, premature atrial and ventricular events, oversensing of *P* and T waves, or noise artefacts.

### Artificial intelligence-based detections by the ILR-ECG-A

**Figure 1:**
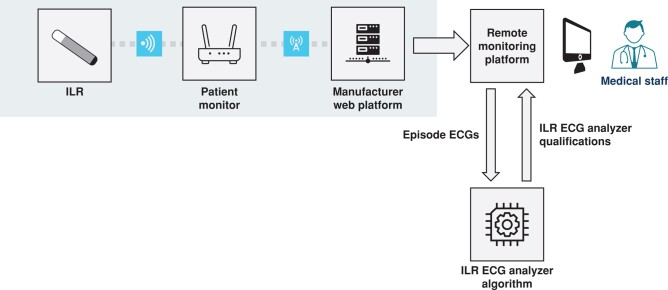
Progression of an ILR event with the addition of the ILR-ECG-a algorithm to the diagnostics. As is usual with RM, the event emitted by the device is transmitted to the manufacturer’s Internet-based platform via the Patient Monitor. From there, the event is transmitted to a RM Platform interfaced with ILR-ECG-A. The event is sent to the algorithm, which adds its diagnostic results before the final transmission to the caregiver.

ILR-ECG-A, an AI-based algorithm, was developed to reclassify episodes recorded by the ICM, with a view of limiting the rate of FP detections. This algorithm uses the ICM signals as input and detects either a ‘Normal Rhythm’ or a list of abnormal events, classified as ‘Asystole’, ‘Bradycardia’, ‘AT/AF’, ‘VT’ (‘ventricular tachycardia’), ‘Artefact’ (uninterpretable signals), or ‘Unspecified Abnormality’. This algorithm comes with a suggestion to healthcare professionals to review in priority every episode not diagnosed as ‘Normal Rhythm’ with the same level of importance, as it is optimized to identify as few abnormal episodes as possible with this diagnosis. It is compatible with the transmission files of the ICM and can be interfaced with the RM platform. This allows access to the ECG signals with the highest accuracy and the transmission of its results to the interface used by the caregiver to follow patients remotely (*Figure 1*). To classify the episodes, the AI-based algorithm automatically uses the settings of asystole, bradycardia, and tachycardia intervals programmed during ICM implantation. The algorithm was developed by a combination of expert features, which include morphological and frequential analyses of sensed QRS and P waves. For example, in addition to the pattern recognition applied to the Lorenz plot included in the ICM algorithms, the acceleration of the heart rate and the related time span between subsequent identifiable rhythms is computed as a feature. This allows us a better differentiation atrial tachycardias from normal sinus tachycardias. An improved QRS detection algorithms using an Optimized Knowledge-Based method^11^ also allowed for better algorithmic interpretations of Lorenz plots. To these expert features, a neural network was added to provide additional automatic features. The underlying architecture is a 1D Convolutional Neural Network (CNN), employing shortcut connections in a manner similar to the Residual Network architecture, as described by Hannun et al.^12^ It has six output classes instead of twelve, corresponding to the first six classes detected by ILR-ECG-A (The ‘Unspecified Abnormality’ output corresponding to the absence of the other six classes). The activations of the penultimate layer are then concatenated to expert features, to extend the information provided by these features. The resulting features are used as input of six machine learning classifiers, which compute scores for each of the possible diagnoses, and classify signal according to the outputs of the classifiers and fixed thresholds determined at the training stage.^13^

### Training methodology of the ILR-ECG-A

**Figure 2:**
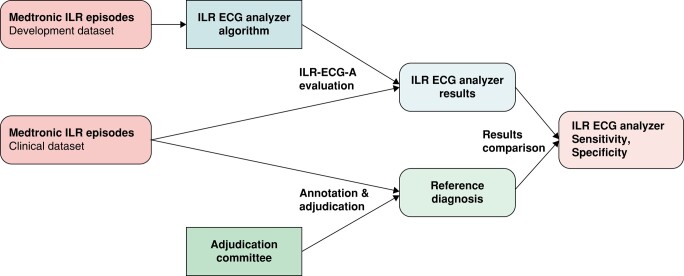
Training of the AI-based ILR-ECG-a, and its evaluation, using an independent dataset, as described in the ‘methods’.

A development dataset of 3405 ICM episodes diagnosed by a panel of 9 expert cardiac electrophysiologists was used to train and validate the algorithm (*Figure 2*). These episodes were collected from 870 patients of 22 European medical centres using the Implicity™ platform to follow their patients. They were selected to have a high variety of ICM episode types: these episodes were selected so that at most 5 episodes per patient and per ICM diagnosis could be selected. As such, the episodes were balanced with respect to the ICM diagnosis. On each of these episodes, the experts annotated the start and end of each diagnosis on the ECG trace. Using a subset of these episodes with expert adjudication, six machine learning algorithms (XGBoost) were trained to qualify the samples as sequentially ‘Artefact or not Artefact’, ‘Asystole or not Asystole’, ‘Bradycardia or not Bradycardia’, ‘AT/AF or not AT/AF’, ‘VT/ventricular fibrillation (VF) or not VT/VF’ and ‘Normal Rhythm or not Normal Rhythm’. The evaluation of each of these classifiers successively on any episode returns a qualification of this episode according to each qualification type. These six algorithms were all trained on binary classification tasks, with the previously described features as input, to which the output of previously trained classifiers, and with the binary label corresponding to their specific task as an optimization target. A hyper-optimization had been performed for each of the six algorithms, with the training dataset, sequentially, to determine their optimal hyper-parameters, and the optimal sequence of algorithms (i.e. the order in which the six algorithms were trained and evaluated).

These qualifications were converted into a final diagnosis by the ILR-ECG-A algorithm using the following rules:

- If any of the abnormal qualification are fulfilled, the final diagnosis is the exhaustive list of abnormal qualification fulfilled for the episode
- Else, if the ‘Normal Rhythm’ qualification was fulfilled, the final diagnosis is ‘Normal Rhythm’
- Else, the final diagnosis was ‘Unspecified Abnormality’

As such, ILR-ECG-A algorithm is conservative on the ‘Normal Rhythm’ diagnosis, as the final diagnosis of an episode can only be ‘Normal Rhythm’ if no abnormality was detected by ILR-ECG-A in any part of the ECG trace.

The performances of the trained algorithms were evaluated on a subset of the development dataset (of patients not included in the training of the algorithm) using the endpoints described in the ‘Study endpoints’ section. The overall expected performance for the algorithm was a sensitivity over 90%, and a specificity over 60%. The performance target set for each individual label was that no Asystole, Bradycardia, or VT would be misdiagnosed as Normal Rhythm by ILR-ECG-A. The fixed thresholds used to classify signals were obtained by optimizing for these expected performances on the validation dataset. The algorithms reached these performance targets on the validation dataset, before the collection and evaluation of this study.

### Episode selection

**Figure 3:**
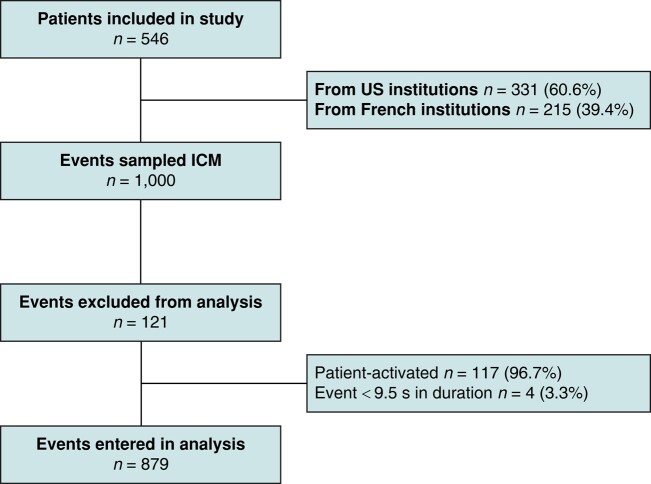
Patient selection and flow of events from the initial inclusion of a predetermined sample of 1000 to the final inclusion of 879 events in the analysis.

To test the ILR-ECG-A algorithm’s performance in a variety of arrhythmias, we sampled unique episodes of each type of arrhythmia transmitted by the patients. When multiple episodes of the same type were transmitted by the same patient, a single sample was retained for the analysis, using a uniform random sampling among the episodes of the same patient and type, to prevent a cluster effect. This data collection method was designed to sample the widest variety of abnormalities and discard redundant episodes. We expected this method to result in a low within-patient correlation among signals, conferring sufficient statistical power to our analysis. This episode selection method selected 1000 episodes for analysis (*Figure 3*). All patient-activated episodes were excluded from the analysis, as the ILR-ECG-A algorithm, which implements the rules used by ICM to detect abnormal episodes, does not reclassify these signals. Events <9.5 s in duration, too short for analysis by the algorithm, were likewise excluded.

### Study endpoints

This study was designed to evaluate the ability of the ILR-ECG-A algorithm to decrease the rate of FP events recorded by Reveal ICM. As such, this study evaluated the ability of the ILR-ECG-A algorithm to detect FP events (i.e. diagnose these episodes as ‘Normal Rhythm’), while misdiagnosing few true positive (TP) episodes—‘Abnormality’—as ‘Normal Rhythm’. Therefore, this study uses a binary endpoint to describe the results of ILR-ECG-A. ‘Artefact’ episodes are defined in this study as episodes where the presence of a non-cardiac signal distorts the ECG enough to prevent medical interpretation. They were then not clearly identifiable as TP or FP events, as it was not possible in the scope of this study to associate them with real patient events. They then constituted a third category, to be analyzed separately from ‘Normal Rhythm’ and ‘Abnormality’. Events were collected by medical centres different from those where the machine learning classifier was trained and selected as described earlier. Therefore, no patient included in this study had episodes that had been used to train or validate the algorithm. The same events were analyzed by an independent adjudication committee (AC) and by the AI-based algorithm. The AC included five experienced cardiac electrophysiologists (Appendix 1), who did not participate in the review of the algorithm’s training data, and who had no access to the patients’ clinical information. They examined each ECG recording and classified them as (i) ‘Abnormality’ (asystole, bradycardia, AT/AF, VT/VF, or other abnormalities), (ii) ‘Normal (sinus) Rhythm’, or (iii) ‘Artefact’, considering the settings of the ICM as the only rule for the events’ classification. Each event was reviewed by two members of the AC and, in case of disagreement, was adjudicated by the Chairman or in a consensus meeting of the Committee. The AC diagnoses were considered as the reference for the evaluation of the manufacturer’s and Implicity’s algorithms.

The ILR-ECG-A algorithm diagnoses were compared with the AC adjudications as a binary classification, excluding ‘Artefacts’ for primary endpoints. The successful or unsuccessful identification of ‘Normal Rhythm’ and ‘Abnormality’ events by the AI-based algorithm was classified as TP_AI_, FP_AI_, True Negative (TN_AI_) or False Negative (FN_AI_). The sensitivity of the ILR-ECG-A algorithm was the proportion of arrhythmic episodes not classified as ‘Normal Rhythm’, calculated as TP_AI_/(TP_AI_ + FN_AI_). Its specificity was the proportion of FP diagnoses by the ICM reclassified as ‘Normal Rhythm’ by the algorithm, calculated as TN_AI_/(FP_AI_ + TN_AI_). The sensitivity and specificity of the ILR-ECG-A algorithm were the primary endpoints of the study. In the context of this study, only signals which were diagnosed as ‘Abnormality’ (positive) by ICMs were available.

### Statistical analyses

No adjustment for multiplicity was made. A single device may have transmitted multiple events. Hence, a patient represents a cluster of personal signals. To minimize a within-cluster/patient correlation of the binary endpoint, the episodes were selected as described earlier. Therefore, the within-patient correlation among signals was assumed to be very low, and independence among signals was assumed in the primary analysis. In presence of multiple signals per patient, a generalized estimating equation (GEE) model assuming a compound symmetry correlation structure was fitted to account for signal correlation within each patient, to verify this assumption. The confidence intervals (CI) were calculated with the Clopper-Pearson exact test, assuming independence between episodes.

The statistical calculations were made, using the SAS software (SAS Institute, Cary, NC). Binary endpoints were estimated along with 95% CI. Tests were performed at the 0.05, two-sided, α-level of significance.

## Results

The mean age of the 546 patients included in this study, of whom 331 (60.6%) underwent implants in the United States and 215 (39.4%) in Europe, was 68.0 ± 17.2 years. The ICM models included 455 (83.3%) LNQ11, 87 (16.0%) REVEAL XT 9529 and 4 (0.7%) REVEAL DX 9528. *Figure 3* summarizes the event-sampling procedure. Of the 1000 episodes sampled (mean = 1.6 ± 0.8/patient), 117 patient-activated and 4 lasting <9.5 s were excluded from the analysis. All the 546 patients had at least one episode included in the analysis. *Table 1* lists the diagnoses made by the AC vs. the ICM for the 879 remaining episodes. Since >1 abnormal rhythm might have been identified in a single event, by the AC or by ILR-ECG-A, the overall ‘Abnormality’ event count was inferior to the sum of the count of episodes annotated with each specific abnormality type. The AC annotated more AT/AF episodes than the ICM devices, as among the 241 episodes classified as ‘Tachy’ by ICM devices, 154 (63.9%) were diagnosed as AT, i.e. AT/AF by the AC.

**Table 1:** Episodes and diagnoses made by the AC and by the ICM, without and with the ILR-ECG-a algorithm

Events diagnoses	By the AC	By the ICM	
		Without ILR-ECG-A	With ILR-ECG-A
Normal rhythm	283 (32.2)	Not applicable	229 (26.1)
Abnormality	516 (58.7)	879 (100)	577 (65.6)
Artefact	80 (9.1)	Not applicable	91 (10.3)
**Abnormality details**			
AT or AF	370 (42.1)	313 (35.6)	451 (51.3)
Asystole	90 (10.2)	208 (23.7)	98 (11.1)
Bradycardia	58 (6.6)	117 (13.3)	63 (7.2)
Other	28 (3.2)	Not applicable	Not applicable
VT or VF	10 (1.1)	241 (27.4)	18 (2.0)
Unspecified abnormality	Not applicable	Not applicable	31 (3.5)

Values are numbers (%) of observations

The AC identified 516 episodes as ‘Abnormality’ and 283 as ‘Normal Rhythm’. The sensitivity and specificity were calculated for the overall sample, and for each ICM model and event type subgroups. A GEE model was fitted for each endpoint and ensured the validity of the assumption of independence between episodes, thus validating the use of Clopper-Pearson exact 95% CI.

### Study endpoints

The overall sensitivity of the ILR-ECG-A algorithm, i.e. the proportion of arrhythmic events which were not classified as ‘Normal Rhythm’ by the AI-based algorithm, was 98.6% (97.2%—99.5%) (superior to the 90% objective). The sensitivity measured by event type is presented in *Table 2A*. A detailed analysis of the FN events, performed to verify the detection of all serious events, and the associated comments by the AC, are shown in *Table 3*. None of these 7 FN episodes was considered diagnostically unambiguous by the AC, they were all diagnosed as Normal Rhythm by one annotator before being adjudicated as Other or AT/AF by the AC. Among these False Negatives, three had been identified as AT/AF by the ICM, three as Tachy, and one as Asytole. The sensitivities were consistent in subgroup analyses by event types (*Table 2A*), by territories (USA vs. Europe) and device models (see Supplementary Material, Supplementary material online, *Table S1*).

**Table 2:** A. Sensitivity and B. Specificity of the ILR-ECG-A algorithm

	A. Sensitivity	(n/N)
**Overall analysis**	98.6 [97.2–99.5]	(509/516)
**By AC event type**		
AT/AF	98.7	(365/370)
Asystole	100.0	(90/90)
Bradycardia	100.0	(58/58)
Other	92.9	(26/28)
VT	100.0	(10/10)
Artefact	92.5	(74/80)
**Abnormalities + artefacts**	97.7 [96.09–98.71]	(582/596)

	B. Specificity	(n/N)
**Overall analysis**	76.0 [70.6–80.8]	(215/283)
**By ICM event type**		
AT/AF	75.0	(89/120)
Asystole	85.7	(54/63)
Bradycardia	73.7	(42/57)
VT	67.4	(29/43)

Table A, the sensitivity is firstly computed for all ‘Abnormality’ events. Then, the Table presents the same sensitivity statistics computed for each particular ‘Abnormality’ event (as diagnosed by the AC). In the last line, the sensitivity on ‘Abnormality’ and ‘Artefact’ events was computed considering ‘Artefacts’ as ‘Abnormality’, as ILR-ECG-A device suggests healthcare professionals using it to consider ‘Artefact’ and all other ‘Abnormality’ outputs with the same level of importance.

B, the specificity is firstly computed for all ‘Normal Rhythm’ events. Then, the table presents the same specificity statistics on the episodes diagnosed with a particular event type by the ICM device. Values are percentages (95% CI).

**Table 3:** FN events detected by the ILR-ECG-a algorithm

Diagnoses		Additional AC comments
By the ICM	Adjudicated by AC	
Tachy Tachy	AT/AF AT/AF	While these events appeared normal, the clockwise regularity of the rhythm at a 400-ms cycle length favoured the diagnosis of AT
Asystole Tachy AT/AF	AT/AF AT/AF AT/AF	These events were very noisy and their analysis was most challenging. Nevertheless, they appeared to be normal rhythm or AT
AT/AF	Other	P waves are visible in a type I second degree atrioventricular block periodicity, therefore not abnormal and classified as ‘Other’.
AT/AF	Other	The baseline rhythm was normal with brief ‘pauses’ consistent with mild sinus node dysfunction, classified as ‘Other’.

AC, adjudication committee

The overall specificity of the ILR-ECG-A algorithm (*Table 2B*), i.e. the proportion of FP classifications by the ICM reclassified as ‘Normal Rhythm’ by the algorithm was 76.0% (95% CI: 70.6—80.8) (superior to the 60% objective). In the analysis by ICM event type, the lowest specificity of the algorithm (29/43–67.4%) was on the VT label. As among episodes diagnosed as VT by the ICM, many were normal sinus tachycardia and AT. These events are particularly difficult to differentiate on ICM traces, especially when the atrial activity is not visible on the ECG and/or the start and end of the episode were not recorded as part of the episode. For this reason, ILR-ECG-A misdiagnosed multiple Tachycardia ICM FP as AT instead of Normal Rhythm (for a normal sinus tachycardia) in such cases. The proportion of FP classifications by ICM reclassified as ‘Normal Rhythm’ by the algorithm cannot be described by AC event type, as the only FP classification available to the AC was ‘Normal Rhythm’ without further details.

**Figure 4:**
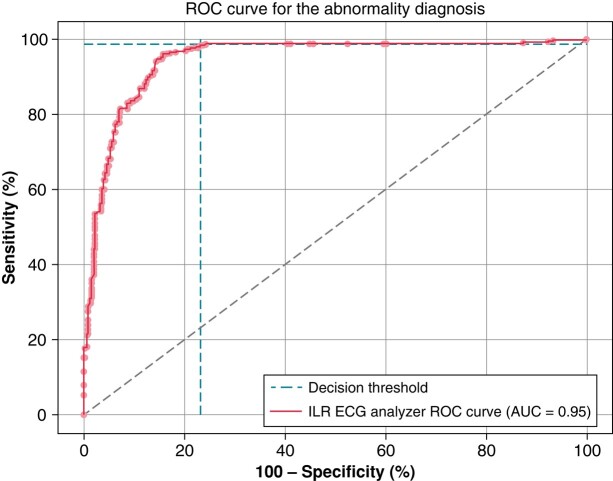
This ROC-curve was plotted for the ‘abnormality’ decision criteria of ILR-ECG-a. The ‘Decision Threshold’ lines display the Sensitivity and Specificity with the threshold used by the algorithm—which correspond to the results Overall Analysis line of *Table 2*.

To better visualize the trade-off between sensitivity and specificity, the receiver operating characteristic curve (ROC-curve) of ILR-ECG-A was computed for the ‘Abnormality’ score, which is to classify signals as either ‘Abnormality’ or ‘Normal Rhythm’ (*Figure 4*). The figure displays the decision thresholds lines, which intersect the ROC-curve at the threshold used by ILR-ECG-A trained algorithm and then corresponds to the overall sensitivity and specificity of the algorithm.

**Figure 5:**
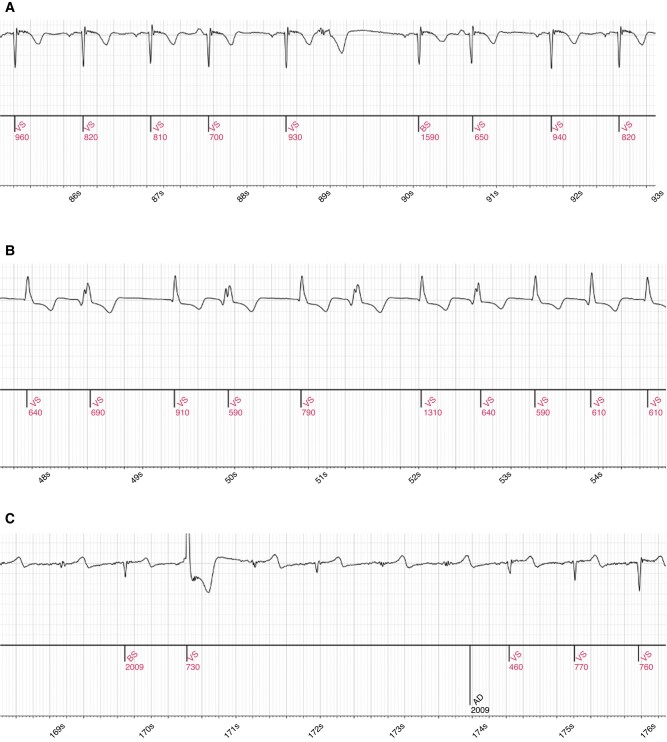

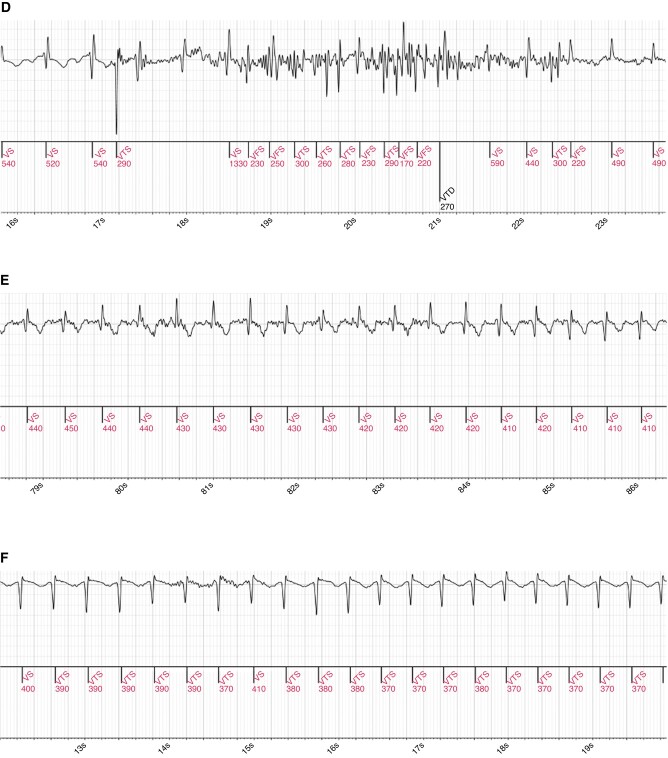
Excerpts of ECG episodes reclassified by ILR-ECG-a, representative of several situations that occurred in this study. (A) ICM diagnosis: AT/AF; ILR-ECG-A diagnosis: Normal Rhythm; AC adjudication: Normal Rhythm. (B) ICM diagnosis: AT/AF; ILR-ECG-A diagnosis: AT/AF; AC adjudication: AT/AF. (C) ICM diagnosis: Asystole; ILR-ECG-A diagnosis: Normal Rhythm; AC adjudication: Normal Rhythm. (D) ICM diagnosis: VT; ILR-ECG-A diagnosis: Normal Rhythm; AC adjudication: Normal Rhythm. (E) ICM diagnosis: VT; ILR-ECG-A diagnosis: Normal Rhythm; AC adjudication: AT/AF. (F) ICM diagnosis: VT; ILR-ECG-A diagnosis: AT/AF; AC adjudication: Normal Rhythm.

Six examples of signals included in this study are presented in *Figure 5*, with their diagnosis by the AC, the ICM, and ILR-ECG-A. The *Figure*  *5A, C* and *D* are TNAI for ILR-ECG-A (TN_AI_), the *Figure 5B* is a TP_AI_, the *Figure 5E* is a FN_AI_, which corresponds to the fourth line of *Table 3*. and the *Figure 5F* is a FP_AI_.

## Discussion

This international study showed that ILR-ECG-A machine learning algorithm led to a correct reclassification of 76.0% of FP episodes, attributable to the filtering of the ICM episodes. Moreover, the algorithm sensitivity was 98.6%, and no critical episode, i.e. asystole, bradycardia, or VT, was identified as ‘Normal Rhythm’ by the device.

Although RM decreases the need for on-site evaluations, management of associated transmissions consumes healthcare resources, with limited reimbursement. The volume of transmissions that trained professionals need to interpret has grown by several orders of magnitude in the past three decades and continues to increase.^14^ False positives are a source of increasing frustration and current proposed solutions include changes from the device nominal settings,^14^ with no evaluated impact on sensitivity. An AI system, such as the algorithm evaluated in this study, is an alternate strategy that can be directly connected to the ICM data transmissions and integrated in the clinician workflow, helping healthcare professionals improve their interpretation of alerts, especially when the volume of data increases. Moreover, AI algorithms may support the medical team focus on fewer ‘actionable’ signals without missing important events. In comparison with a change in the device settings, this allows an equivalent decrease in the rate of FPs to be reviewed, while keeping the filtered episodes in store in the RM platform, should further examinations or investigations be needed. Importantly, the AI algorithm should not filter TP episodes. We found a 98.6% overall sensitivity of the AI-based algorithm, due to 7 FN events (*Table 3*). Although this study focused on the detection of FP events as ‘Normal Rhythm’, the clinical usefulness of such AI algorithm could be analyzed further by evaluating its performance as a multi-class classifier. This would assess its capability to identify the correct list of abnormalities shown in a given ICM episode, in addition to correctly identifying them as abnormal.

In one prior study of AI applied to ICM diagnosed AF episodes, the most common reason for FP AF events was premature atrial contractions, and the algorithm reduced AF FP events by 39.5% to 66.4%, depending on the cohort. However, that study differs significantly from ours since it analyzed total episodes labelled as AF by ICM devices i.e. 1500 episodes for 425 patients. In comparison, we sampled one episode per patient per ICM diagnosis, and included a wider range of diagnoses i.e. asystole, bradycardia and VT, which represent an important proportion of FP alerts emitted by ICM.

A study using a single-lead ECG combined with a machine learning algorithm demonstrated the possibility to improve the early identification of patients at risk for AF-induced cardiomyopathy.^15^ In a recent publication, it has been demonstrated the ability of a AI-based algorithm to predict the risk of AF from sinus rhythm recordings.^16^ Thus, AI-based algorithms are probably the future of atrial fibrillation diagnosis, either for ICM or for wearable devices.^17^ This study focused on the Reveal DX, XT and LINQ I (Medtronic) devices, with limited analytical capabilities; the new generation of ICM, such as the Linq II (Medtronic), introduces an algorithm based on AI, improving sensitivity. The main challenge in the near future will be to convince healthcare professionals to trust AI algorithms in their clinical practice, without delegating their responsibility.^18^ To achieve this goal, AI algorithms must integrate transparency, traceability,^19^ and explicability^20^ The use of AI in medicine is no more a novelty. It is used in cardiology and in particular in rhythm analysis, such as ECG^21^ and atrial fibrillation.^16,22^

The Implicity platform is designed as an agnostic tool, able to display all manufacturers devices data with the same ergonomics. In addition, alerts are filtered and sorted by severity. This approach has been shown to reduce reviews by 57%.^23^ For this reason, we can expect a reduction in the workload of the medical team. By extension, costs can be expected to be reduced, as health professionals can focus their activities on relevant alerts requiring early intervention.

### Study limitations

As mentioned in its FDA 510(k) approval, ILR-ECG-A interpretation results are not intended to be the sole means of diagnosis. It is offered to physicians and clinicians on an advisory basis only in conjunction with the physician's knowledge of ECG patterns, patient background, clinical history, symptoms, and other diagnostic information.

The study included only 16% and 0.7% of patients with a Reveal XT or Reveal DX. These proportions were obtained by randomly sampling patients followed with the Implicity™ platform on 2020–2011, and are expected to be representative of the Medtronic device repartition as that time, but it means that the level of proof for ILR-ECG-A sensitivity and specificity is lower on older devices than on the more recent Reveal LINQ I. Additionally, the ethnicity and gender of the patients included in this study were not collected. Hence, potential bias of the algorithm results across ethnicity and gender are not evaluated in this study and is only mitigated by the variety of medical centres included in this study.

One hundred and seventeen patient-activated episodes were excluded from the analysis, as the ILR-ECG-A algorithm, which implements the rules used by ICM to detect abnormal episodes, does not reclassify these signals. Further development of the algorithm could allow it to provide clinical value with a reclassification of these episodes.

Data collection in each patient selected a single type of episode, potentially introducing a selection bias in favour of rare kinds of episodes. This procedure was used to minimize the intra-patient correlations and expose the algorithm to a variety of events. A preliminary study evaluating the proportion of episodes diagnosed as Normal Rhythm in an unbiased dataset was conducted and showed that 33% of all episodes (including episodes without ECG trace and patient-activated episodes) were reclassified as Normal Rhythm by ILR-ECG-A.^24^

## Conclusion

Given that ICM implant volume coupled to RM is expected to grow in upcoming years, the novel ILR-ECG-A AI-based algorithm that filters nearly 100% of FP ICM events and can be easily integrated into current workflow provides an opportunity to alleviate the heavy device clinic workload associated with ICM management.

## Supplementary Material

euad375_Supplementary_Data

## Data Availability

The data underlying this article cannot be shared publicly due to the European GDPR (UE 2016/679). The data will be shared on reasonable request to the corresponding author in accordance with anonymization process and GDPR. All patients had granted their written approval to contribute the data at the time of RM activation, in particular for its use for the purpose of research & development activities, including design of algorithms. All data were de-identified to ensure the protection of personal health data, according to the European regulation and French reference methodology (MR-004), and in accordance with the HIPAA de-identification requirements in the US. Since this was a retrospective analysis of prospectively collected clinical data in real-life practice, this study was exempt from reviews and approvals by the institutional review boards of the participating institutions, the post-processing is conducted in accordance with the European ‘General Data Protection Regulation’ (UE 2016/679—article 5) and FDA regulations in the US.

## Appendix

The AC was composed of the following board-certified cardiac electrophysiologists:

**Chairman:** Etienne Aliot, MD, FHRS, Centre Hospitalier Régional Universitaire, Nancy, France

**Members:**

Nicolas Sadoul, MD PhD, Centre Hospitalier Régional Universitaire, Nancy, France

Hugues Blangy, MD, PhD, Centre Hospitalier Régional Universitaire, Nancy, France

Laurence Guédon-Moreau, MD, Centre Hospitalier Universitaire, Lille, France

Claude Kouakam, MD, Centre Hospitalier Universitaire, Lille, France
