# Supplementary material for: Improved diagnostic performance of insertable cardiac monitors by an artificial intelligence-based algorithm
Source: Europace. 2024 Jan 3;26(1):euad375. doi: 10.1093/europace/euad375 (PMC10787483; doi:10.1093/europace/euad375)
Supplement: euad375_Supplementary_Data [file euad375_supplementary_data.pdf]

Supplementary Material – Table 1. Sensitivity and Specificity of the ILR-ECG-A algorithm by territory and device model

	A. Sensitivity	(n/N)	Specificity	(n/N)
**Overall analysis**	98.6 [97.2-99.5]	(509/516)	76.0 [70.6-80.8]	(215/283)
**By Territory**				
United States	98.5	(329/334)	69.3	(124/179)
Europe	98.9	(180/182)	87.5	(91/104)
**By Device Model**				
LNQ11	99.3	(413/416)	79.5	(174/219)
REVEAL DX 9528	100.0	(2/2)	100.0	(2/2)
REVEAL DX 9529	95.9	(94/98)	62.9	(39/62)
